# Artificial Intelligence and Large Language Models: Editorial Reflections

**DOI:** 10.4274/TJAR.2025.252355

**Published:** 2025-12-22

**Authors:** Zekeriyya Alanoğlu, Serkan Tulgar, Alper Kılıçaslan, Özlem Selvi Can

**Affiliations:** 1 Washington University in St. Louis, School of Medicine, Department of Anesthesiology St. Louis, United States; 2 Bahçeşehir University Faculty of Medicine, Göztepe Medical Park Hospital, Department of Anesthesiology and Reanimation, İstanbul, Türkiye; 3 Necmettin Erbakan University Faculty of Medicine, Department of Anesthesiology and Reanimation, Konya, Türkiye; 4 Ankara University Faculty of Medicine, Department of Anesthesiology and Reanimation, Ankara, Türkiye

The *Turkish Journal of Anaesthesiology and Reanimation* reflects current developments in anaesthesiology through the scientific work that emerges organically from the field, rather than through predefined thematic or special issues. Each issue therefore represents a snapshot of the academic and clinical interests shaping the discipline at a given time. As a result, certain subjects may assume greater visibility within individual issues. While research on regional anaesthesia featured prominently in a recent issue, artificial intelligence (AI) has attracted similar attention in the present volume. This pattern should be understood as a reflection of evolving priorities within anaesthesiology, rather than as the outcome of editorial direction.

Such convergence allows the editorial board to step back from thematic labeling and instead engage in critical appraisal. Examining emerging trends, placing them in appropriate context, and encouraging scholarly discussion on their relevance to clinical practice and education form a core part of this editorial role. In the present issue, Dost et al.^1^ contribute a narrative review that maps the current and potential applications of AI in anaesthesiology and reanimation, offering a broad clinical and conceptual overview. Alongside this, an original study examines the performance of an AI based model in an assessment format modeled on international anaesthesiology examinations.^2^ Although distinct in design and scope, both articles provide a forward-looking perspective and invite reflection on the changing role of AI in clinical work and medical training.

The journal’s position on the use of AI in scientific publishing, particularly large language models (LLMs), is grounded in transparency, methodological rigor, and scientific responsibility. These tools are neither inherently advantageous nor intrinsically problematic; their value depends on how they are used and reported.^3^ Submissions involving LLMs are therefore expected to clearly define their role and limitations, and to meet standards that safeguard academic integrity and reproducibility.

The use of LLMs in defining a research question or shaping study methodology fundamentally undermines the originality of a study, even when the final version of the manuscript is written independently and appears novel in the literature, and is therefore not recommended. Similarly, assigning statistical analyses directly to LLMs is not currently considered appropriate; however, as contemporary models increasingly rely on the infrastructure of established professional statistical software, this perspective may evolve provided that outputs are independently validated. The increasing availability of AI-supported and commercially established online statistical platforms also suggests that AI-assisted data analysis may soon become more common, reinforcing the need for transparency and validation.

By contrast, the use of LLMs for language revision is supported and encouraged, provided that the human character and stylistic integrity of the text are preserved. We do not intend to revive earlier debates on this issue; when transparently declared in the acknowledgements, employing such tools for linguistic refinement may be both practical and beneficial. However, delegating the writing of an entire manuscript to LLMs—an approach we have unfortunately encountered in submissions to the journal—may result in hallucinated content, fabricated references, and text that is uniform, repetitive, and lacking the practical reasoning characteristic of human intelligence. In addition, authors should carefully consider issues of data security and confidentiality, as sharing unpublished data, original analyses, or proprietary material with AI platforms may inadvertently expose sensitive information, increase the risk of misuse, or compromise intellectual ownership.

Beyond our views on the use of AI and LLMs in clinical research and reporting, it is also appropriate to address expectations and observations looking ahead. Much like the surge of Coronavirus Disease 2019 (COVID-19)-related publications during the pandemic, studies exploring the use of AI across different areas of medicine have recently attracted considerable attention. This rapid expansion has led to a noticeable inflation within the literature, making critical appraisal and scientific selectivity increasingly important. Just as the readability and clinical relevance of COVID-19-related publications declined once the acute phase of the pandemic subsided, a similar trajectory may await AI-related manuscripts when AI becomes a routine component of daily clinical practice rather than an emerging novelty. For this reason, the journal will not favor studies that merely repeat existing knowledge, lack clear clinical relevance, or represent short-lived trends likely to fade after brief discussion. Instead, priority will be given to work that openly addresses its limitations, offers new perspectives for future research, questions the role of AI, or challenges it to improve. Studies conducted with appropriate methodology and high scientific quality, including those engaging with AI at a conceptual or philosophical level, will be welcomed.

Popularity alone will not justify overlooking scientific standards.
